# Analyse des Stellenwertes von „eLearning“ in der Augenheilkunde und Evaluierung einer „eLearning-App“

**DOI:** 10.1007/s00347-020-01100-x

**Published:** 2020-04-17

**Authors:** E. Grabowski, F. Alten, J. Termühlen, P. Heiduschka, V. Brücher, N. Eter, C. R. Clemens

**Affiliations:** Universitäts-Augenklinik Münster, Domagkstr. 15, 48149 Münster, Deutschland

**Keywords:** Computergestützter Unterricht, Interaktives Lernen, Smartphone, Applikation Software, Evaluation, Computer-assisted Instruction, Interactive learning, Smartphone, Applikation software, Evaluation

## Abstract

**Hintergrund:**

Ziel der Studie war die Analyse des Stellenwertes von „eLearning“ im Lern- und Fortbildungsverhalten von Augenärzten in Deutschland sowie die Bewertung der Akzeptanz einer neuen „eLearning“-Anwendersoftware (App).

**Material und Methoden:**

Ophthalmologische Weiterbildungsassistenten und Fachärzte wurden im Rahmen von Fortbildungsveranstaltungen mittels eines Fragebogens nach ihren Fortbildungsaktivitäten befragt. Des Weiteren erfolgte nach Vorstellung und Anwendung einer „eLearning-App“ eine strukturierte Bewertung.

**Ergebnisse:**

Es nahmen 149 Augenärzte an der Befragung teil. Während der überwiegende Teil der Kollegen (74,3 %) analoge Fachzeitschriften wöchentlich oder monatlich zur Weiterbildung nutzt, verwenden 45,9 % der Augenärzte digitale Printmedien (Bücher, Zeitschriften, Artikel) sowie 46,5 % Fachbücher in gedruckter Form. Lediglich 35 % der Befragten bilden sich über Online-Fortbildungsplattformen, z. B. digitale Kurse (CME-Kurse) oder Portale zum Abrufen aufgezeichneter Vorträge, fort. Die Nutzung der angebotenen „eLearning-App“ ging insgesamt mit einer positiven Akzeptanz einher; 91,7 % der befragten Kollegen würden diese Form der interaktiven Lernvermittlung weiterempfehlen.

**Diskussion:**

Trotz fortschreitender Digitalisierung in allen Lebensbereichen nimmt „eLearning“ als Lernmedium in der ophthalmologischen Fort- und Weiterbildung weiterhin einen geringen Stellenwert ein. Interessanterweise ergab die Bewertung der App-Nutzer eine hohe Benutzerakzeptanz, unabhängig von Alter oder Arbeitsbereich.

## Abstract (en)

**Background:**

The aim of the study was to analyze the importance of e‑learning in the learning and training behavior of ophthalmologists in Germany and to evaluate the acceptance of a new e‑learning user software (app).

**Material and methods:**

Ophthalmological residents and specialists were asked about continuing education activities by means of a questionnaire during continuing education events. Furthermore, a structured evaluation was carried out after the presentation and application of an e‑learning app.

**Results:**

A total of 149 ophthalmologists took part in the survey. While the majority of colleagues (74.3%) used specialist journals weekly or monthly for further education, 45.9% of ophthalmologists used digital print media (books, journals, articles) and 46.5% used specialist books in printed form. Only 35% of the interviewees used online training platforms, e.g. digital courses (CME courses) or portals for retrieving recorded lectures. The use of the offered e‑learning app was generally accompanied by a positive acceptance. Of the interviewed colleagues 91.7% would recommend this form of interactive learning.

**Discussion:**

Despite a progressive digitalization in all areas of life, e‑learning continues to play a minor role as a learning medium in ophthalmological advanced training. Interestingly, the evaluation of app users showed a high level of acceptance, regardless of age or field of work.

„eLearning“ steht für das Lernen mithilfe verschiedener elektronischer oder digitaler Medien und bietet die Möglichkeit des individualisierten Lernens unabhängig von Ort und Zeit [2, 9]. Dennoch findet „eLearning“ nur zögerlich Einsatz im Fort- und Weiterbildungsspektrum im Fachbereich der Augenheilkunde.

In der Augenheilkunde gibt es einige Online-Angebote, die den Augenarzt im Alltag unterstützen können [12]. Hierbei handelt es sich insbesondere um Nachschlagewerke, Videosammlungen oder digitale Diagnostikanwendungen, wie z. B. eine Anwendersoftware (App) zum Auslösen des optokinetischen Nystagmus. Viele dieser Angebote fallen nicht unter den Oberbegriff des „eLearnings“, da sie keinen strukturierten oder interaktiven Erwerb von ophthalmologischem Wissen bieten. Hier ist das Angebot gegenwärtig begrenzt. Dabei steigert eigene Interaktion die Lernmotivation und führt dazu, Inhalte besser und nachhaltiger zu verinnerlichen. Eine interaktive Lernumgebung entsteht beispielsweise, wenn der Nutzer am Ende eines Lernabschnittes sich seines Lernerfolgs anhand von Testfragen selbst vergewissern kann und das „eLearning“-Programm automatisch die weiteren Lerninhalte am Lernerfolg oder -misserfolg gezielt ausrichtet. Interaktion kann eine „eLearning“-Plattform auch in Gestalt einer Diskussions- und Austauschmöglichkeit mit anderen Lernenden und Lehrenden bieten. Abgesehen von Fachzeitschriften und Online-Plattformen (z. B. CME-Fortbildungen), stehen in der Augenheilkunde nur vereinzelte Online-Angebote, oft ausschließlich in englischer Sprache zur Verfügung, die als „eLearning-Tool“ interaktiv und einer zielgerichteten Didaktik folgend Inhalte vermitteln [5, 7]. Trotz zahlreicher guter Ideen und Programmansätze scheitern die Projekte oftmals aufgrund zeitintensiver und personalaufwendiger Erstellung, hoher Entwicklungskosten und komplizierter urheber- und nutzungsrechtlicher Fragen [1]. Zweifelsohne eignet sich das Feld der Augenheilkunde zum selbstständigen Lernen, angeleitet durch elektronische Medien. Aus dieser Überlegung resultierte die Entwicklung einer fallbasierten „eLearning“-App für die eigenständige Fort- und Weiterbildung (Abb. 1). Ziel der Studie war die Analyse des Stellenwertes von „eLearning“ im Lern- und Fortbildungsverhalten von Augenärzten in Deutschland sowie die Bewertung der Akzeptanz der neu entwickelten App.

**Figure 1:**
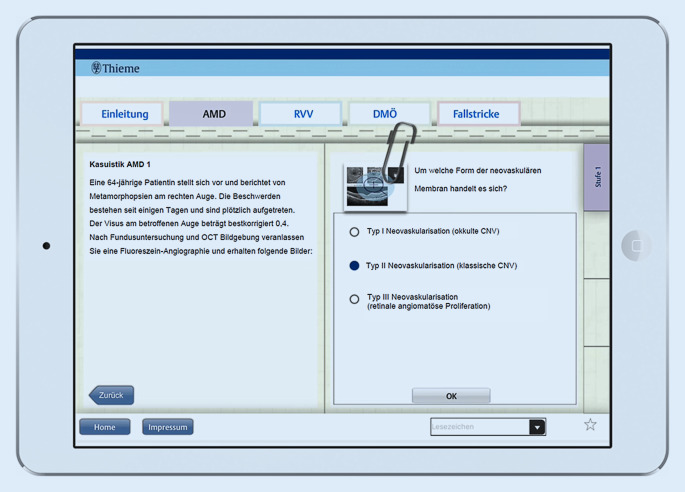

## Methoden

### Das Design der „eLearning-App“

Wir entwickelten eine Anwendersoftware mit dem Schwerpunkt der Netzhautdiagnostik und Befundung von optischen Kohärenztomographieaufnahmen (OCT). Hierzu wurden sowohl typische als auch außergewöhnliche Fälle aus der retinologischen Sprechstunde zusammengestellt und professionell für die Präsentation in einer „eLearning“-App aufgearbeitet. Jeder Fall beinhaltet eine Patientenanamnese und entsprechende Netzhautaufnahmen unterschiedlicher Bildgebungsmodalitäten (Fundusfoto, OCT, Fluoreszeinangiographie etc.) Der Fallbeschreibung schließt sich eine Frage mit 3 Antwortmöglichkeiten an. Die Fälle gliedern sich in 4 Themengebiete: 1. altersabhängige Makuladegeneration, 2. retinale Venenverschlüsse, 3. diabetische Netzhauterkrankungen und 4. Fallstricke (Abb. 1). Die Kasuistiken wurden vor dem Hintergrund des aktuellen Wissensstandes beleuchtet, und therapeutische Optionen wurden nach den Leitlinien und Stellungnahmen der augenärztlichen Fachgesellschaften kommentiert. Um das Lernverhalten der jeweiligen Teilnehmer zu charakterisieren und die Anwendersoftware zu evaluieren, wurden alle Nutzer aufgefordert, einen Fragebogen zu beantworten. Die Datenerhebung erfolgte im Rahmen von Fortbildungsveranstaltungen sowohl in Papierform als auch elektronisch. Erfragt wurden Alter, Geschlecht, derzeitiger Ausbildungsstand (Weiterbildungsassistent, Facharzt), Beschäftigungs- (Forschung, Niederlassung, Klinik) und Tätigkeitsbereich (konservativ, operativ). Zusätzlich wurden die Nutzer nach ihren Erfahrungen mit der App befragt: Ist das Design benutzerfreundlich und gestaltet sich die Handhabung intuitiv? Ist die Qualität der Bilddarstellung ausreichend? Werden alltägliche Problemstellungen des Augenarztes realitätsnah abgebildet? Werden mit dem Lernformat die Lernziele erreicht? Haben Sie mithilfe der App neues Wissen erlernt? Hat Sie das Lernformat zum Lernen motiviert? Würden Sie diese Lernform Kollegen weiterempfehlen?

Die App wurde vom Georg Thieme Verlag mit finanzieller Unterstützung der Firma Bayer gestaltet. Weder der Georg Thieme Verlag noch die Firma Bayer hatten Einfluss auf Design und Durchführung der vorgestellten Studie. Der Inhalt der Publikation spiegelt ausschließlich die Meinungen der Autoren, nicht die der genannten Projektpartner wider. Die App ist kostenfrei im passenden App-Store verfügbar.

### Statistische Analyse des Fragebogens

Die statistische Auswertung erfolgte mit Microsoft® Excel® 2010 (Microsoft Corporation, Redmond, WA, USA) und GraphPad® PRISM® (Version 8) (Graphpad Software, Inc. San Diego, CA, USA). Die Studienpopulation wurde durch deskriptive statistische Standardmessungen beschrieben. Kontinuierliche Variablen, von denen eine Normalverteilung angenommen wurde, werden als Mittelwert ± Standardabweichung (SD) dargestellt. Bei nichtlinearen Skalen wurde eine nichtlineare Ranganalyse unter Berechnung des Spearman-Korrelationskoeffizienten durchgeführt.

## Ergebnisse

**Figure 2:**
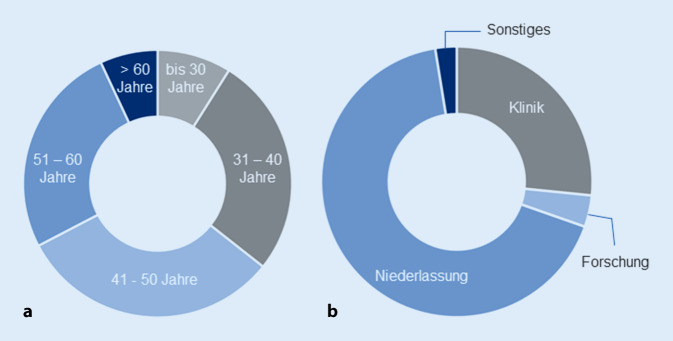

Es nahmen 149 Personen an der Befragung teil (50,3 % Frauen). *Alle Fragebögen waren vollständig auswertbar*; 57 % der Befragten waren zwischen 41 und 60 Jahre alt (Abb. 2a); 91 Personen waren konservativ ausgerichtet und größtenteils niedergelassen tätig (Niederlassung 66,6 %, Klinik 27,2 %) (Abb. 2b).

Die Umfrage ergab, dass Fachzeitschriften in gedruckter Form das am häufigsten angewendete Fortbildungsmedium in der wöchentlichen bzw. monatlichen Nutzung darstellt; 74,3 % der befragten Kollegen bilden sich mittels analoger Fachzeitschriften wöchentlich oder monatlich fort, gefolgt von digitalen Printmedien (Bücher, Zeitschriften, Artikel) in 45,9 % der Fälle und Fachbücher in gedruckter Form in 46,5 %. Lediglich 35 % nutzen Online-Fortbildungsplattformen, z. B. digitale Kurse (CME-Kurse) oder Portale zum Abrufen aufgezeichneter Vorträge (Tab. 1). Eine Auswertung der Ergebnisse in Abhängigkeit des Tätigkeitsbereichs ergab keine nennenswerten Unterschiede.

**Table 1:** 

Fortbildungsmodalität	Nutzung wöchentlich oder mehrfach pro Woche (%)	Nutzung ca. monatlich (%)	Nutzung 1‑mal pro Jahr oder seltener (%)
Fachbücher	21,5	25,0	53,5
Fachzeitschriften	27,8	46,5	25,7
Digitale Artikel	24,6	21,3	54,1
Online Fortbildungsplattformen (z. B. digitale Kurse, aufgezeichnete Vorträge)	11,7	23,3	65,0

**Figure 3:**
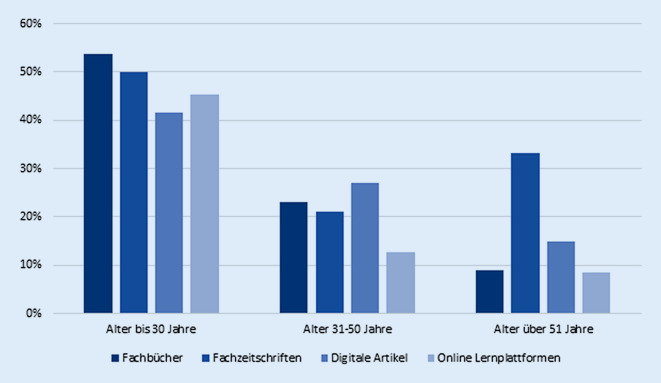

Betrachtet man die Altersgruppen separat, stellt sich folgendes Bild dar. Digitale Lernmedien werden insbesondere von jüngeren Kollegen (<30 Lebensjahre) in Kliniktätigkeit genutzt. Dabei finden Online-Plattformen (45,5 %) und digitale Artikel (41,7 %) während der Facharztausbildung häufig Anwendung. Im Vergleich dazu gaben Nutzer zwischen 31 und 50 Jahren für alle genannten Lernmedien eine seltenere Anwendung an, lediglich 12,7 % nutzen Online-Plattformen und weniger als 30 % digitale Artikel zur Weiterbildung. Erfahrene Kollegen (>51 Lebensjahre) bilden sich insbesondere mittels Fachzeitschriften in gedruckter Form weiter (33,3 %), gefolgt von Artikeln in digitaler Form (15,0 %) und Online-Fortbildungsplattformen (8,6 %) (Abb. 3).

Keiner der Teilnehmer hatte „eLearning“-Erfahrung in der Augenheilkunde. Unabhängig vom Alter beurteilten über 90 % der Befragten, dass das Design der Anwendersoftware benutzerfreundlich und die Handhabung einfach und intuitiv war. Insgesamt war die Qualität der Bilddarstellung ausreichend, und mit den dargestellten Fällen wurden alltägliche Problemstellungen des Augenarztes realitätsnah abgebildet. Es gaben 90,8 % der App-Nutzer an, durch das Format zum Lernen motiviert zu werden, und 86,8 % hatten durch die Fallpräsentation und die anschließende Erklärung neues Wissen erlernt. Der Gesamteindruck der Anwendersoftware wurde positiv bewertet (Schulnote: 1,67 ± 0,72).

**Figure 4:**
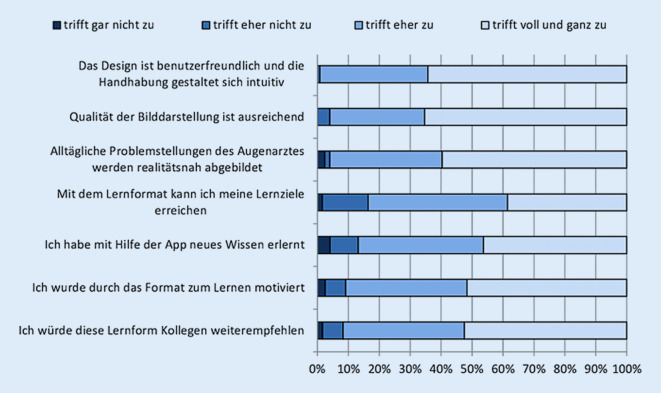

Die Umfrage ergab, dass das Lernformat mit einer hohen Benutzerakzeptanz einhergeht und gute Möglichkeiten liefert, Patientenbeispiele ausreichend darzustellen und Problemstellungen realitätsnah abzubilden. Insgesamt würden 91,7 % der Kollegen die „eLearning“-App als Lernmedium weiterempfehlen (Abb. 4).

## Diskussion

Grundsätzlich nimmt „eLearning“ in der augenärztlichen Fort- und Weiterbildung im Vergleich zu konventionellen Lernmethoden einen geringen Stellenwert ein, während im „eLearning“-Sektor insgesamt betrachtet ein schnelles Wachstum zu verzeichnen ist [4]. So zeigt beispielsweise eine Auswertung der von der Ärztekammer Westfalen-Lippe anerkannten medizinischen Fortbildungsveranstaltungen seit der ersten Erfassung der Kategorien I und K („eLearning“ und „blended Learning“) 2014 einen Zuwachs dieser Angebote um 113 %, verglichen mit einem Zuwachs aller anderen anerkannten Veranstaltungen um lediglich 17 % [6]. Während die Anwendung von Online-Artikeln und Lehrbüchern im Bereich der Augenheilkunde weiterhin die am häufigsten genutzten Lernmedien darstellen, werden von der Ärztekammer Westfalen-Lippe bisher keine ophthalmologischen Fortbildungen im Bereich „eLearning“ und „blended Learning“ erfasst. Unsere Arbeit zeigt, dass eine interaktive Anwendersoftware von Benutzern als gewinnbringendes „eLearning“ Instrument bewertet wird.

**Figure 5:**
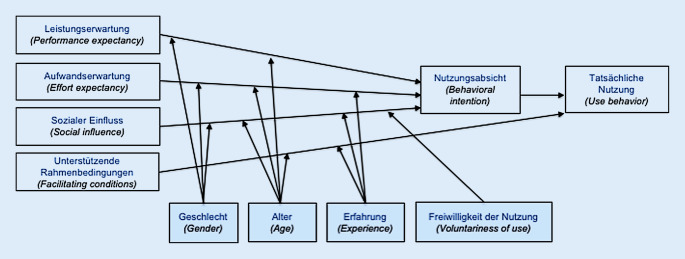

Für viele Anwender bedeutete die Nutzung der angebotenen „eLearning“-App die erste Auseinandersetzung mit einem solchen Lernformat. Eine intuitive Anwendung und ein übersichtliches Design sind für den Erfolg essenziell. Neben einem möglichst hohen Maß an Benutzerfreundlichkeit sollte das Lernmedium zusätzlich ein positives Nutzungserlebnis bieten [13]. Grundsätzlich beruht die Einstellung bzw. Akzeptanz für eine neue Technologie auf mehreren Faktoren und ist nicht von lediglich einer Komponente abhängig. Venkatesh et al. entwickelten ein Technologieakzeptanzmodell (Unified Theory of Acceptance and Use of Technology [UTAUT-Modell]), um das individuelle Nutzerverhalten für eine neue Technologie erklären bzw. vorhersehen zu können [14] (Abb. 5).

Um eine möglichst hohe Akzeptanz für eine neue Technologie zu erreichen, werden 4 Hauptkriterien beschrieben, die als Stellschrauben für die Beeinflussung der Nutzer dienen:

1. Leistungserwartung („performance expectancy“),
2. Aufwandserwartung („effort expectancy“),
3. sozialer Einfluss („social influence“),
4. unterstützende Rahmenbedingungen („facilitating conditions“).

Die Leistungserwartung wird als der Grad verstanden, von dem der Anwender glaubt, dass die angewandte „neue“ Technologie seine Arbeitsleistung verbessern wird. Sie stellt den stärksten Prädikator zur Verhaltensabsicht dar. Die Vorteile der App-Anwendung für die persönliche Arbeit des Augenarztes sind offensichtlich: leichte Zugänglichkeit, mühelose Anwendung und hohe Relevanz für das eigene Tätigkeitsfeld. Die Auswertung zeigt, dass die Anwender einen hohen Nutzen in der „eLearning“-App sehen; >80 % haben mit dem Lernformat neues Wissen erlernt und ihre Lernziele erreicht.

Die Aufwandserwartung stellt die erwartete Anstrengung für den Anwender dar, welche mit der Verwendung der „neuen“ Technologie verbunden ist. Vor Beginn und innerhalb der Anfangsphase der Anwendung hat dieser Faktor einen Einfluss. Der Effekt nimmt mit zunehmender Nutzung ab. In Bezug auf die angebotene „App“ scheint die Anstrengung für den Anwender niedrig. Alle Nutzer kamen von Beginn an mühelos mit der Handhabung zurecht und beurteilten das Design als benutzerfreundlich.

Der soziale Einfluss definiert den Grad der Wahrnehmung, ob Personen aus dem Umfeld zu einer Nutzung der „neuen“ Technologie raten. Dieser Aspekt wurde im Fragebogen nicht direkt abgefragt, allerdings impliziert die Aussage „Ich würde die App Kollegen weiterempfehlen“ einen prädiktiven Wert für die Verbreitung der Anwendersoftware; >90 % der Befragten stimmten dieser Aussage „voll und ganz“ zu.

Unterstützende Rahmenbedingungen zielen auf die Erwartung einer vorhandenen organisatorischen und technischen Infrastruktur hin, innerhalb derer eine „neue“ Technologie genutzt wird. Da inzwischen sowohl App-Anwendungen als auch mobile onlinefähige Tablets und Smartphones weite Verbreitung und Akzeptanz gefunden haben, stellt dieser Aspekt keine Hürde dar.

Geschlecht, Alter und Erfahrung spielen eine zu vernachlässigende Rolle auf die Nutzungsabsicht der angebotenen Anwendersoftware. Interessanterweise wurde in der vorliegenden Untersuchung die „App“ unabhängig vom Alter akzeptiert. Möglicherweise ist die Aussicht auf eine signifikante Nutzensteigerung bei minimalem Aufwand ausschlaggebend für dieses Ergebnis.

Lernerfolg durch „eLearning“ wird in der wissenschaftlichen Literatur unterschiedlich bewertet. Chumley-Jones et al. zeichnen in ihrem umfangreichen Review-Artikel ein heterogenes Bild. Zahlreiche Vergleichsstudien zum Lernerfolg zeigten keinen erkennbaren Vorteil für das „eLearning“ [4]. Einige Arbeiten bescheinigen dem „eLearning“ allerdings einen vergleichbaren Lernerfolg mit weniger Zeitaufwand gegenüber dem Einsatz traditioneller Lernmethoden [3, 9]. Eine Schwierigkeit stellt hier sicherlich die nur eingeschränkte Vergleichbarkeit der Studien dar. Ferner ist der Bereich des „eLearnings“ auch unmittelbar der rasanten technologischen Entwicklung unterworfen, sodass Studien zu „eLearning“-Tools beispielsweise aus den 2000er-Jahren gegenwärtig nur noch begrenzte Aussagekraft besitzen.

Smeds et al. untersuchten den Effekt einer ausbildungsbegleitenden App während der chirurgischen Rotation bei britischen Medizinstudenten und analysierten die Auswirkung auf die Prüfungsleistung der Studenten. Hierzu wurden fallbasierte Fragen erstellt und auf freiwilliger Basis über eine mobile App an die Studenten verteilt. Die Prüfungsergebnisse der App-Nutzer wurden schließlich mit den Ergebnissen der Nicht-Nutzer verglichen. Die App-Nutzer schnitten deutlich besser in den Abschlussprüfungen ab als diejenigen, die die begleitende Lern-App nicht genutzt haben [11]. Lewis et al. beschreiben in ihrer Arbeit mobile Anatomieausbildungstools und weisen auf die Hauptstärke dieser Anwendungen hin, die in der interaktiven dreidimensionalen Funktionalität liegt. Durch einfache Berührung werden virtuelle dreidimensionale Modelle gedreht bzw. gezoomt und anatomische Strukturen identifiziert, ein- und ausgeblendet. Dies wird häufig durch Quizfunktionen und Multiple-Choice-Frage-Tests begleitet. Derartige Lernanwendungen bieten dem Lernenden einen erweiterten visuellen und interaktiven Zugang zum Lerninhalt, der von konventionellen Lernmedien nicht abgebildet werden kann [8].

Für die einen Autoren ist „eLearning“ in erster Linie kostenintensiv und bringt keinen nachweisbaren Mehrwert. Für andere Autoren stellt es das Lernmedium der Zukunft dar [4]. Insgesamt gibt es bis dato nicht ausreichend Daten, um zu beurteilen, ob „eLearning“-Maßnahmen in der Medizin einen messbaren, positiven Effekt auf das Behandlungsverhalten von im Gesundheitswesen tätigen Personen oder auf die Behandlungsergebnisse aufseiten der Patienten hat [10]. An diesen Kriterien sollten sich Untersuchungen zum „eLearning“ zukünftig orientieren.

Die vorgestellte Studie ist nicht ohne Limitationen. Alle 4 von Venkatesh beschriebenen Hauptfaktoren konnten nicht prospektiv abgefragt werden, da die App-Anwendung vor der Beantwortung des Fragebogens erfolgten musste.

Gegenwärtig spielt „eLearning“ in der augenheilkundlichen Fort- und Weiterbildung eine untergeordnete Rolle. Unsere Arbeit zeigt, dass durch eine interaktive Lernvermittlung mittels einer Anwendersoftware die Bedingungen für eine hohe Akzeptanz erfüllt werden. Die Kombination aus Portabilität und Zugänglichkeit in Verbindung mit der Bandbreite an medizinischen Inhalten bedeutet, dass eine solche Lernform wahrscheinlich eine zentrale Rolle in der zukünftigen Fort‑/Weiterbildung spielen wird.

## Fazit für die Praxis

- „eLearning“ ist in der augenheilkundlichen Fort- und Weiterbildung nicht weit verbreitet.
- Die vorgestellte Anwendersoftware wurde in vielerlei Hinsicht positiv bewertet.
- Die Vorteile (flexibler und niederschwelliger Zugang sowie Interaktion und direktes Feedback) lassen eine weitere Verbreitung von „eLearning“-Tools in der Augenheilkunde und in der augenheilkundlichen Fort- und Weiterbildung erwarten.
